# Supplementary material for: Electrochemotherapy in Vulvar Cancer and Cisplatin Combined with Electroporation. Systematic Review and In Vitro Studies
Source: Cancers (Basel). 2021 Apr 21;13(9):1993. doi: 10.3390/cancers13091993 (PMC8122585; doi:10.3390/cancers13091993)
Supplement: Supplementary file 1 [file cancers-13-01993-s001.zip › cancers-1176337-supplementary.pdf]

cancers

MDPI

Systematic Review

Electrochemotherapy in Vulvar Cancer and Cisplatin Combined with Electroporation. Systematic Review and In Vitro Studies

Anna Myriam Perrone [1,2,3], Gloria Ravegnini [4,*], Stefano Miglietta [2,4,5], Lisa Argnani [6], Martina Ferioli [7,8], Eugenia De Crescenzo [1,3], Marco Tesei [1,2], Marco Di Stanislao [1,3], Giulia Girolimetti [2,3,5], Giuseppe Gasparre [2,3,5], Anna Maria Porcelli [2,4,5,9], Francesca De Terlizzi [10], Claudio Zamagni [2,11], Alessio Giuseppe Morganti [2,7,8,†] and Pierandrea De Iaco [1,2,3,†]

[1] Division of Oncologic Gynecology, IRCCS—Azienda Ospedaliero-Universitaria di Bologna, 40138 Bologna, Italy; myriam.perrone@aosp.bo.it (A.M.P.); eugenia.decrescenzo@studio.unibo.it (E.D.C.); marco.tesei@aosp.bo.it (M.T.); marco.distanislao@studio.unibo.it (M.D.S); pierandrea.deiaco@unibo.it (P.D.I.)
[2] Centro di Studio e Ricerca delle Neoplasie Ginecologiche (CSR), University of Bologna, 40138 Bologna, Italy; stefano.miglietta2@unibo.it (S.M.); giulia.girolimetti3@unibo.it (G.G.): giuseppe.gasparre3@unibo.it (G.G.); annamaria.porcelli@unibo.it (A.M.P.);claudio.zamagni@aosp.bo.it (C.Z.); alessio.morganti2@unibo.it (A.G.M.);
[3] Department of Medical and Surgical Sciences (DIMEC), University of Bologna, 40138 Bologna, Italy
[4] Department of Pharmacy and Biotechnology, University of Bologna, 40126 Bologna, Italy
[5] Center for Applied Biomedical Research, Alma Mater Studiorum-University of Bologna, 40138 Bologna, Italy
[6] Institute of Hematology, Department of Experimental, Diagnostic and Specialty Medicine, University of Bologna, 40138 Bologna, Italy; lisa.argnani@unibo.it
[7] Radiation Oncology, IRCCS Azienda Ospedaliero-Universitaria di Bologna, 40138 Bologna, Italy; martina.ferioli4@unibo.it
[8] Department of Experimental, Diagnostic and Specialty Medicine, University of Bologna, 40138 Bologna, Italy
[9] Interdepartmental Center for Industrial Research Life Sciences and Technologies for Health, Alma Mater Studiorum-University of Bologna, 40064 Ozzano dell'Emilia, Italy
[10] Scientific & Medical Department IGEA S.p.A., 41012 Carpi (Mo), Italy; f.deterlizzi@igeamedical.com
[11] Oncologia Medica Addarii, IRCCS Azienda Ospedaliero-Universitaria di Bologna, Via Albertoni 15, Bologna, 40138, Italy
* Correspondence: gloria.ravegnini2@unibo.it
† Equally contributors.

Supplementary

Citation: Perrone, A.M.; Ravegnini, G.; Miglietta, S.; Argnani, L.; Ferioli, M.; De Crescenzo, E.; Tesei, M.; Stanislao, M.D.; Girolimetti, G.; Gasparre, G.; et al. Electrochemotherapy in Vulvar Cancer and Cisplatin Combined with Electroporation. Systematic Review and In Vitro Studies. *Cancers* **2021**, *13*, 1993. https://doi.org/10.3390/cancers13091993

Received: 24 March 2021
Accepted: 19 April 2021
Published: 21 April 2021

Publisher's Note: MDPI stays neutral with regard to jurisdictional claims in published maps and institutional affiliations.

Copyright: © 2021 by the authors. Submitted for possible open access publication under the terms and conditions of the Creative Commons Attribution (CC BY) license (http://creativecommons.org/licenses/by/4.0/).

cancers MDPI

Table S1. Palliative chemotherapy and radiotherapy in vulvar carcinomas. 1

				Radiotherapy			Chemotherapy		
**Study**	**Setting**	**Number of pts**	**Number of VC treated with palliative intent**	**Type**	**Response**	**OS**	**Drug**	**Response**	**OS**
Wagenaar et al, 2001 [57]	Locally advanced VC	25	25	/	/	/	BLM, methotrexate, and CCNU	PR: 48.0% CR: 8.0%	Median: 7.8 months
Hruby et al, 2000 [62]	Recurrent VC	26	NR	EBRT+/- surgery +/- chemo	PR: 40.0% CR: 20.0%	5-year: 46% (1) 0% (2)	Different regimens	/	/
Tewari et al, 1999 [61]	Advanced/recurrent VC	11	6 Previously irradiated	BRT +/- EBRT (3)	NR	All pts treated for palliation died of disease (8-64 months)	/	/	/
Benedetti-Panici et al, 1993 [59]	Advanced VC	21	21	/	/	/	CSP, BLM, and methotrexate	PR (T): 9.5% CR (T): 0.0%	NR
Durrant et al, 1990 [58]	Locally advanced VC	31	31	/	/	/	BLM, methotrexate, and CCNU	PR: 48.4% CR: 9.7%	NR
Prempree et al, 1984 [60]	Recurrent VC	21	13	BRT +/- EBRT	31.0%	5-year: 0% in VC > 5 cm	/	/	/

Legend: (1) confined to the vulva; (2): extending beyond the vulva; (3) : one patient developed perineal necrosis and one patient developed rectovaginal fistula. BRT: brachitherapy; BLM: bleomycin; CCNU: lomustine; CR: complete response; CSP: cisplatin; EBRT: external beam radiotherapy; PR: Partial response; NR: not responder OS: overall survival; T: tumor; VC: vulvar cancer. 2 3 4
